# Vanishing Bile Duct Syndrome Preceding the Diagnosis of Hodgkin Lymphoma

**DOI:** 10.14309/crj.0000000000000336

**Published:** 2020-03-02

**Authors:** Chimaobi Anugwom, Grete Goetz, Mohamed Hassan

**Affiliations:** 1 Division of Gastroenterology, Hepatology and Nutrition, University of Minnesota, Minneapolis, MN

## Abstract

Vanishing bile duct syndrome is an acquired liver disease characterized by the progressive destruction and loss of intrahepatic bile ducts. It usually signifies end-stage cholestatic liver disease and is characterized by ductopenia on liver biopsy specimen. We present a case of vanishing bile duct syndrome as the presenting symptom in a patient with Hodgkin lymphoma with subsequent improvement after adequate treatment of the lymphoma.

## INTRODUCTION

Vanishing bile duct syndrome (VBDS) is an acquired liver disease characterized by the progressive destruction of intrahepatic bile ducts and frequently signifies end-stage cholestatic liver disease, with little to no consequence on liver synthetic function except in protracted and severe cases.^1^ The cumulative result is the pathologic diagnosis of ductopenia, which is confirmed on an adequate liver biopsy specimen. It has been associated with multiple medications: bacterial, viral, and parasitic infections; ischemic conditions affecting the biliary tree, and neoplastic diseases. Typically, it presents as a complication after the diagnosis of the primary pathology.^2^ We present a case of VBDS as the presenting symptom in a patient with Hodgkin lymphoma.

## CASE REPORT

A 27-year-old woman with idiopathic intracranial hypertension, obesity, and asthma was seen in the hospital with mild abdominal cramps and itching and a 2-day history of watery stools and vomiting in the preceding days. She had no fever, recent travel, or sick contacts. Her daily medications include acetazolamide, daily omeprazole and albuterol. She does not smoke and drinks approximately 3 alcoholic beverages per week. She was hemodynamically stable on presentation, and her physical examination was significant for mild epigastric tenderness and scleral icterus. Her initial blood tests revealed alanine transaminase of 272 U/L, aspartate transaminase of 188 U/L, alkaline phosphatase of 239 U/L, and total bilirubin of 6 mg/dL with a direct bilirubin of 5.4 mg/dL. Given these laboratory findings, an abdominal ultrasound with Doppler was obtained, which suggested fatty liver disease but no evidence of biliary or gallbladder disease. Additional imaging studies included an unremarkable hepatobiliary iminodiacetic acid scan and a magnetic resonance cholangiopancreatography that showed no focal liver masses, mildly distended gall bladder, and no calcified gallstones, with normal intrahepatic and extrahepatic bile ducts. Exhaustive infectious disease workup was negative for hepatitis C, hepatitis B, *Cytomegalovirus*, human immunodeficiency virus, Epstein-Barr virus, and herpes simplex as well as autoimmune hepatitis and primary biliary cholangitis. Her home medications were stopped at the time of admission, and her liver tests improved slightly. Her liver injury was believed to be due to acetazolamide, and she was discharged with close follow-up.

**Figure 1.**
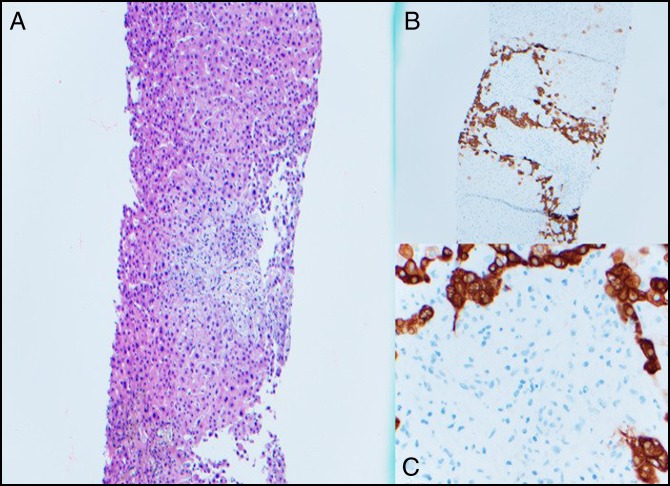
Liver biopsy showing the absence of native bile duct using hematoxylin and eosin stain (A) and cytokeratin 7 (B and C).

She was seen a week later in clinic, and her liver tests had worsened with an aspartate transaminase of 695 U/L, alanine transaminase of 809 U/L, alkaline phosphatase of 1,312 U/L, and total bilirubin of 13 mg/dL, with direct bilirubin of 11.4 mg/dL. She was otherwise clinically stable but complained of mild itching. She was readmitted into the hospital where her liver test results continued to increase. At this time, a transjugular liver biopsy was obtained and portal pressures were measured at 3 mm Hg. Throughout her time of evaluation, her international normalized ratio remained normal and her serum albumin ranged between 2.9 and 3 g/dL. The liver biopsy pathology showed an absence of bile ducts with ductular reaction suggestive of VBDS (Figure 1).

**Figure 2.**
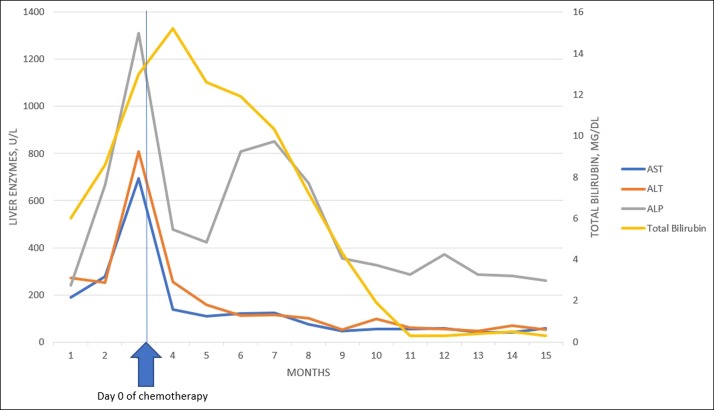
Trend of liver enzymes with chemotherapy for Hodgkin lymphoma. ALP, alkaline phosphatase; ALT, alanine transaminase; AST, aspartate transaminase.

As soon as this diagnosis was made, a careful review of her medical records was carried out and a chest x-ray obtained previously was noted to have mild mediastinal widening. A computed tomography scan of her chest was subsequently obtained, which noted mediastinal lymph nodes. She later underwent a computed tomography-guided anterior mediastinal lymph node biopsy which revealed the diagnosis of Hodgkin lymphoma. She was started on radiotherapy and a combination of high-dose steroids (dexamethasone) with rituximab, gemcitabine, and cisplatin. Her bilirubin and liver enzymes remained significantly elevated before commencing treatment (Figure 2). This regimen was selected because she was deemed not to be a candidate for the typical adriamycin (doxorubicin), bleomycin, vinblastine, and dacarbazine, given her elevated liver tests. She improved on this regimen and was later switched to adriamycin (doxorubicin), bleomycin, vinblastine, and dacarbazine regimen with achievement of remission. She was followed up closely in the hepatology clinic, and within 12 months, her liver tests revealed normalization of bilirubin with stable transaminases (Figure 2). On a follow-up with Neurology, after an exhaustive discussion, she elected not to restart acetazolamide and monitor her idiopathic intracranial hypertension conservatively.

## DISCUSSION

VBDS has been described in the literature to be due to a variety of hepatic insults resulting in the progressive destruction and disappearance of the intrahepatic bile ducts and ultimately, cholestasis. The cumulative result is the pathologic diagnosis of ductopenia, which has been described as a common pathway for most cholestatic diseases.^1,3^ The small ductules of the biliary tree, measuring less than 15 μm, are usually affected, and so hepatobiliary imaging may fail to provide a diagnosis.^4^ VBDS is believed to be an upset in the balance between biliary epithelial cell apoptosis and regeneration, favoring apoptosis with increased expression of B-cell lymphoma-2-associated X-protein and tumor necrosis factor-alpha.^1^ There has been postulations about the presence of immune-mediated injury, not just in the established autoimmune diseases, that involves recognition of antigens on the biliary epithelium, resulting in a cascade of events that ultimately ends in apoptosis and predominantly CD3+ T-cell cytotoxicity.^5^

VBDS can occur because of a myriad of causes which range from graft-vs-host disease to autoimmune diseases to infectious and neoplastic processes (Table 1).^3,6^ There has been documented reports of VBDS presenting as a paraneoplastic phenomenon after the diagnosis of uterine malignancy, Hodgkin, and non-Hodgkin lymphoma.^2,7^ Drug-induced VBDS is also well documented in the literature with antibiotics well represented in this group of inciting events.^8^ Although acetazolamide can cause liver injury, this is rare, related to hypersensitivity, and is usually associated with other immune-allergic features such as fever, rash, and eosinophilia.^9^

**Table 1.**
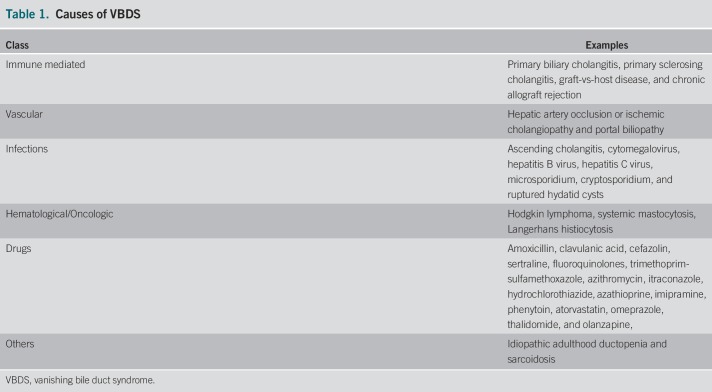
Causes of VBDS

The diagnosis of VBDS requires a high index of suspicion in patients who present with direct hyperbilirubinemia and other clinical and laboratory findings suggestive of cholestasis. The patient's medication history, including over-the-counter medications, is crucial and should be complete. Furthermore, adequate serological evaluation for autoimmune hepatitis and primary biliary cholangitis as well as extensive imaging should be carried out. The final diagnosis is established with an adequate liver biopsy. In an adequate liver biopsy specimen, containing at least 10 portal tracts, ductopenia is defined as the loss of bile ducts in more than 50% of the small portal tracts.^3^ The diagnostic yield can be increased by immunostaining for cytokeratin 7 and 19, as was done in our patient.^10^ CK7 is a low molecular weight cytokeratin. It is a positive marker for atypical ductules such as proliferated ductules and for mature ducts, making it highly sensitive in detecting bile duct tissue. As such, it is used in the diagnostic evaluation of ductopenia as seen in VBDS and Alagille syndrome. In addition, ductular proliferation, which may coexist with duct loss, should be evaluated for. In some cases, no inciting cause of VBDS is found and this entity is termed idiopathic adulthood ductopenia.^3^ Prognosis of VBDS is variable and depends on the balance between biliary epithelial cell apoptosis and regeneration. In patients with unrelenting liver injury, irreversible bile duct loss may ensue, resulting in extensive ductopenia and secondary biliary cirrhosis. In other cases, the insult may be eradicated and biliary epithelial regeneration and clinical recovery can occur over months to years. Idiopathic adulthood ductopenia has a poor prognosis with liver failure occurring in up to 50% of patients.^11^

The management of VBDS primarily involves the removal of the offending medication or treating the causative autoimmune disease or malignancy. Ursodeoxycholic acid acts as a choleretic, anti-inflammatory, immune-modulating, and cytoprotective medication with antiapoptotic properties and has been shown to be beneficial in patients with VBDS.^12,13^ In severe cases and in patients with cirrhosis, liver transplantation may be an option.^14^ Our patient had significant improvement in liver enzymes and resolution of jaundice with adequate treatment of Hodgkin lymphoma and has continued to do well.

VBDS has been described as a known complication of Hodgkin lymphoma and is frequently diagnosed after the lymphoma is identified. In our patient, the diagnosis of VBDS was made before the detection of lymphoma and this pattern of events is quite unusual. Successful treatment of the malignancy in these cases may result in improvement of the liver disease.

## DISCLOSURES

Author contributions: C. Anugwom wrote the manuscript. G. Goetz revised the manuscript for intellectual content. M. Hassan approved the final manuscript and is the article guarantor.

Financial disclosure: None to report.

Informed consent was obtained for this case report.
